# Supplementary figures and images for: Chitosan Fibers Loaded with Limonite as a Catalyst for the Decolorization of Methylene Blue via a Persulfate-Based Advanced Oxidation Process
Source: Polymers (Basel). 2022 Nov 27;14(23):5165. doi: 10.3390/polym14235165 (PMC9736373; doi:10.3390/polym14235165)

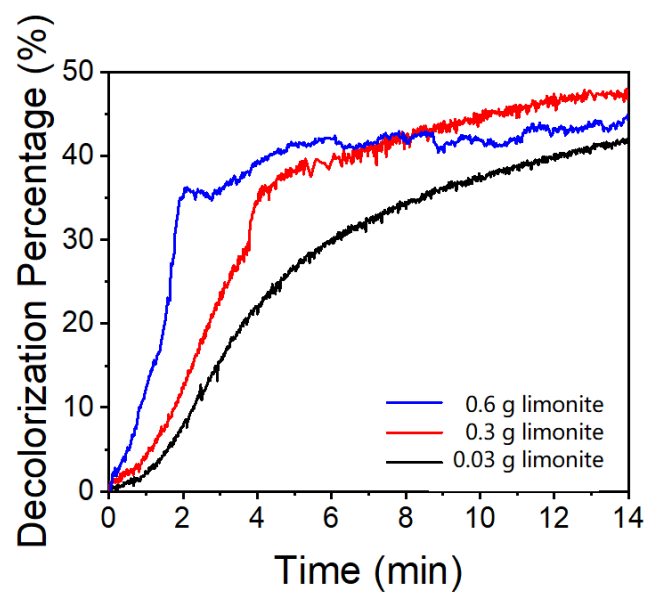

Figure S1. Decolorization kinetics of MB with 4 mM PS using limonite powder (0.03 g, 0.3 g, and 0.6 g).

Supplement: Supplementary file 1 [file polymers-14-05165-s001.zip › polymers-2015706-supplementary.pdf]
